# NF-κB activation in inflammatory breast cancer is associated with oestrogen receptor downregulation, secondary to EGFR and/or ErbB2 overexpression and MAPK hyperactivation

**DOI:** 10.1038/sj.bjc.6603906

**Published:** 2007-08-14

**Authors:** S J Van Laere, I Van der Auwera, G G Van den Eynden, P van Dam, E A Van Marck, P B Vermeulen, L Y Dirix

**Affiliations:** 1 Translational Cancer Research Group, Lab Pathology University of Antwerp, Universiteitsplein 1 and Oncology Center, General Hospital Sint-Augustinus, Oosterveldlaan 24, Wilrijk B2610, Belgium

**Keywords:** inflammatory breast cancer, NF-κB, oestrogen receptor, mitogen-activated protein kinase, EGFR, ErbB2

## Abstract

Activation of NF-κB in inflammatory breast cancer (IBC) is associated with loss of estrogen receptor (ER) expression, indicating a potential crosstalk between NF-κB and ER. In this study, we examined the activation of NF-κB in IBC and non-IBC with respect to ER and EGFR and/or ErbB2 expression and MAPK hyperactivation. A qRT–PCR based ER signature was evaluated in tumours with and without transcriptionally active NF-κB, as well as correlated with the expression of eight NF-κB target genes. Using a combined ER/NF-κB signature, hierarchical clustering was executed. Hyperactivation of MAPK was investigated using a recently described MAPK signature (Creighton *et al*, 2006), and was linked to tumour phenotype, ER and EGFR and/or ErbB2 overexpression. The expression of most ER-modulated genes was significantly elevated in breast tumours without transcriptionally active NF-κB. In addition, the expression of most ER-modulated genes was significantly anticorrelated with the expression of most NF-κB target genes, indicating an inverse correlation between ER and NF-κB activation. Clustering using the combined ER and NF-κB signature revealed one cluster mainly characterised by low NF-κB target gene expression and a second one with elevated NF-κB target gene expression. The first cluster was mainly characterised by non-IBC specimens and IHC ER+ breast tumours (13 out of 18 and 15 out of 18 respectively), whereas the second cluster was mainly characterised by IBC specimens and IHC ER− breast tumours (12 out of 19 and 15 out of 19 respectively) (Pearson *χ*^2^, *P*<0.0001 and *P*<0.0001 respectively). Hyperactivation of MAPK was associated with both ER status and tumour phenotype by unsupervised hierarchical clustering using the MAPK signature and was significantly reflected by overexpression of EGFR and/or ErbB2. NF-κB activation is linked to loss of ER expression and activation in IBC and in breast cancer in general. The inverse correlation between NF-κB activation and ER activation is due to EGFR and/or ErbB2 overexpression, resulting in NF-κB activation and ER downregulation.

Breast cancer is the most frequent cause of cancer death in women worldwide (Key *et al*, 2001) and represents the second leading cause of cancer death among women in the United States (National Center for Chronic Disease Prevention and Health Promotion, 2006) (Key *et al*, 2001; Dumitrescu and Cotarla, 2005; Draper, 2006). Inflammatory Breast Cancer (IBC) is a distinct clinical subtype of locally advanced breast cancer (LABC), with a particularly aggressive behaviour and poor prognosis. Clinically, IBC typically presents with rapidly progressive breast erythema, warmth, oedema and induration (Haagensen, 1956, p. 488). At the time of diagnosis, most patients have axillary lymph node involvement and 1 out of 3 patients have metastasis in distant organs (Kleer *et al*, 2000; Lerebours *et al*, 2005). The characteristic pathology is the invasion of dermal lymphatics by tumour emboli, however, this is only present in 50–75% of the cases (Kleer *et al*, 2000; Low *et al*, 2004; Lerebours *et al*, 2005). Hence, IBC is primarily a clinical diagnosis classified as T4d in the TNM classification of the American Joint Committee on Cancer (Singletary *et al*, 2002). Despite advances in multidisciplinary treatment, the prognosis of IBC is less favourable than of non-IBC, with a 3-year survival of about 40% (Lerebours *et al*, 2005).

Recently, both *in vitro* and *in vivo* experiments have indicated that the biology of IBC has some important differences with the biology of other breast carcinomas. Overexpression of the RhoC GTPase (Van Golen *et al*, 1999; Van Golen, 2003; Kleer *et al*, 2004; Van den Eynden *et al*, 2004) and loss of WISP3 protein expression (Van Golen *et al*, 1999; Kleer *et al*, 2004) are highly correlated with the IBC phenotype. It has been demonstrated that overexpression of the RhoC GTPase is directly and specifically implicated in the production of angiogenic factors by IBC cells (Van Golen *et al*, 2000). In human samples, increased angiogenesis in IBC was evident by both an increased number of microvessels and a higher fraction of proliferating endothelial cells (Colpaert *et al*, 2003), as well as increased expression of several angiogenic growth factors and growth factor receptors in IBC compared to non-IBC (Van der Auwera *et al*, 2004). The specific biology of IBC was further demonstrated by the fact that a molecular signature based on the differential expression of 756 genes is able to separate IBC from non-IBC in an unsupervised hierarchical clustering analysis. The 756 genes-based molecular signature was subjected to a detailed analysis, revealing the presence of several NF-κB target genes and upstream activators of the NF-κB signalling pathway, with strong expression in IBC as compared to non-IBC (Van Laere *et al*, 2005). The NF-κB signature has been validated using qRT–PCR for NF-κB target genes and immunohistochemistry and NF-κB DNA-binding experiments for different NF-κB transcription factors (Van Laere *et al*, 2006b).

The activation of NF-κB in breast cancer has been extensively described in oestrogen receptor negative (ER−) breast tumours and ER− breast cancer cell lines suggesting an important inhibitory crosstalk between both signalling pathways (Biswas *et al*, 2000, 2001, 2004; Zhou *et al*, 2005). This inhibitory cross-talk can be appreciated from the fact that an increase in both NF-κB DNA-binding activity (Nakshatri *et al*, 1997; Pratt *et al*, 2003) and expression of NF-κB target genes like IL8 (Freund *et al*, 2004) coincides with a shift from oestrogen dependence to oestrogen independence in breast cancer. The inverse correlation between ER and NF-κB activity is further supported by the fact that some breast tumours, resistant to the tumoricidal effect of anti-estrogens, become sensitised to apoptosis and show a reduction in NF-κB activity after treatment with oestrogen (Jordan, 2004). This suggests that the proapoptotic effects of oestrogen in these tumours are mediated through inhibition of NF-κB (Jordan, 2004). In our previous study, the significant increase in NF-κB target gene expression was not only linked to the IBC phenotype, but also to the ER status. In addition, a significantly elevated amount of transcriptionally active NF-κB dimers in ER− tumours as compared to ER+ tumours was observed (Van Laere *et al*, 2006b). The fact that IBC is more often ER− as compared to non-IBC is probably one of the reasons for increased NF-κB activation in IBC as compared to non-IBC.

Recent data suggest that all ER− breast tumours arise from ER+ breast cancer cells that stop expressing ER. Proposed mechanisms for the origin of ER− breast cancers include that of pressures being exerted on ER+ cells by oestrogen withdrawal, hypoxia, or overexpression of epidermal growth factor receptor or ErbB2 resulting in MAPK hyperactivation (Creighton *et al*, 2006). Hyperactive MAPK leads to the loss of ER expression. In addition, the indirect activation of NF-κB due to MAPK hyperactivity plays a role in downregulating ER expression (Creighton *et al*, 2006). Recently, the importance of hyperactivated MAPK has been indicated by the fact that an EGFR/ErbB2-induced MAPK signature was able to correctly predict the ER status in four independent data sets with accuracies ranging from 68 to 87% (Creighton *et al*, 2006). This mechanism can also explain the apparent inverse correlation between ER and NF-κB activation.

In the present study, we investigated the activation of the NF-κB transcription factor in breast cancer in terms of ER signalling and the hyperactivation of MAPK due to EGFR and/or ErbB2 overexpression, with special emphasis on the IBC phenotype. We demonstrated that, NF-κB activation is not exclusively limited to IBC but more general to ER− breast tumours. In addition, we demonstrated that IBC is characterised by MAPK hyperactivation in comparison to non-IBC, potentially due to overexpression of EGFR and/or ErbB2. The activation of NF-κB and the frequent ER independency of IBC tumours can be explained in this context. Altogether, these data indicate that NF-κB and MAPK might be therapeutic targets for IBC specifically and more general for ER− breast tumours as well as for breast tumours with acquired resistance against hormonal therapy.

## MATERIALS AND METHODS

### Patients and samples

Tumour samples were obtained from patients with breast adenocarcinoma treated in the General Hospital Sint-Augustinus, Wilrijk, Belgium. Each patient gave written informed consent. This study was approved by the local institutional review board. All samples were stored in liquid nitrogen within 15 min after excision (median delay of 9 min). Breast tumour samples included 17 pretreatment samples of patients with IBC, diagnosed by strictly respecting the criteria mentioned in the TNM classification of the American Joint Committee on Cancer as T4d (Singletary *et al*, 2002). The presence of tumour emboli was, as an isolated pathological finding, not sufficient for the diagnosis of IBC. Of the 20 non-IBC samples, 10 represented LABC (7 T3, 3 T4), four samples represented T2-tumours and six represented T1-tumours. Thirteen patients with non-IBC had pathological axillary lymph node involvement. Baseline clinicopathologic characteristics for the IBC and non-IBC patients from which samples have been used for qRT–PCR analysis and IHC analysis are provided in Table 1.

### RNA isolation, reverse transcription and quantitative real-time RT–PCR

For quantitative Real-Time RT–PCR, RNA was isolated as described before from all samples (Van Laere *et al*, 2005, 2006b). One microgram of RNA from 17 IBC samples and 20 non-IBC samples in total was reverse transcribed into cDNA with random primers (High Capacity cDNA Archive Kit, Applied Biosystems, Foster City, CA, USA). PCR primers and Taqman probes for eight NF-κB target genes (vascular cell adhesion molecule 1 (VCAM1), CC chemokine receptor 5 (CCR5), superoxide dismutase 2 (SOD2), interleukin 15 (IL15), cathepsin B (CTSB), interferon regulatory factor 7 (IRF7), guanylate-binding protein 1 (GBP1), CD48 antigen (CD48)), ER-*α*, ER-*β*and 11 putative ER target genes (PR (PGR), GATA-binding protein 3 (GATA3), mucin 1 (MUC1), x-box binding protein 1 (XBP1), oncogene MYB (MYB), B-cell translocation gene 2 (BTG2), transforming growth factor *β* 3 (TGFb3), Ras-associated protein RAB31 (RAB31), START domain containing 10 (STARD10), hydroxysteroid dehydrogenase 17 *β*4 (HSD17b4) and TRIpartite motif-containing protein 25 (TRIM25)) and two housekeeping genes were purchased as assays-on-demand products for gene expression (Applied Biosystems). Putative ER target genes were selected based upon a constitutive overexpression in ER+ breast tumours in three independent genome–wide breast cancer profiling studies (Abba *et al*, 2005). 18S ribosomal RNA and *β*-actin were used as housekeeping genes to control for reverse transcriptase efficiency, RNA degradation, PCR inhibition and RNA input. Human Universal Reference RNA (Stratagene, La Jolla, CA, USA) was used as calibrator to calculate relative gene expression for the above-mentioned genes. Quantitative real-time RT–PCR was performed on the ABI 7700 Sequence Detector (Applied Biosystems). All PCRs were performed in duplicate. Relative Gene Expression (RGE) was calculated with the 2-ddCt method (Livak and Schmittgen, 2001) as described before (Van Laere *et al*, 2005, 2006b).

### Immunohistochemistry

For NF-κB, EGFR and ErbB2, antibodies purchased from Santa Cruz Biotechnology (Santa Cruz, CA, USA) were used for immunohistochemical (IHC) staining of RelA (clone C-20, sc-372), RelB (clone C-19, sc-226), NFkB1 (clone C-19, sc-1190), NFkB2 (clone K-27, sc-298), cRel (clone B-6, sc-6955), EGFR (clone 1005, sc-03) and ErbB2 (clone F-11, sc-7301). Formalin-fixed, paraffin-embedded tissue sections from 17 IBC tumours and 20 non-IBC tumours were rehydrated through sequential changes of alcohol and distilled water. Antigen retrieval was performed for 30 min in citrate buffer (pH 6) for RelA, RelB, NFkB1, NFkB2 and EGFR at 95°C. For cRel and ErbB2, antigen retrieval was performed for 30 min in Tris–EDTA buffer (pH 9) at 95°C. Sections were incubated for 1 h at room temperature using a dilution of respectively 0.2, 4.0, 1.3, 2.7, 2.0, 8.0 and 4.0 *μ*g ml^−1^. The Dako Envision system on the Dako Cytomation autostainer was used for visualisation of the antibody binding. Tissue sections were counterstained using haematoxylin and mounted for light microscopy. For NF-κB, hot spots with nuclear staining were searched for in each tissue section and within these hot spots a total number of 500 nuclei was counted at a magnification of × 400. Transcriptionally active NF-κB dimers have been determined as described before (Van Laere *et al*, 2006b). The HER2/neu score using the HercepTest (DakoCytomation, Glostrup, Denmark) was interpreted on a 0–3 score (0=no staining or membranous staining in less than 10% of tumour cells; 1=faint or barely perceptible partial membranous staining in more than 10% of tumour cells; 2=weak to complete membranous staining in more than 10% of tumour cells; 3=strong complete membranous staining in more than 10% of tumour cells). Based on this score, the HER2/neu status was determined: Score 0–1 was considered negative, Scores 2 and 3 was considered positive. For EGFR, the same scoring system was used (Van den Eynden *et al*, 2004). Immunohistochemical overexpression of ErbB2 (2+ or 3+) was confirmed by FISH.

For ER quantification, the ER PharmDX (Dako) assay was used. The ER/PR PharmDX assay is an FDA-approved assay, which consists of a cocktail of two mouse monoclonal antibodies to ER (clones 1D5 and ER-2-123), a negative reagent control containing appropriate Ig concentrations equivalent to the primary antibodies, and cell line control slides. Each cell line control slide contains sections of two pelleted formalin-fixed, paraffin-embedded cell lines, which represent a moderate level of ER and PR protein expression (cell line CAMA-1) and a negative cell line (cell line HT-29). Formalin-fixed and paraffin-embedded tissue sections were rehydrated through sequential changes of alcohol and distilled water. Antigen retrieval was performed at 125°C during 5 min in a pressure cooker using Target Retrieval Solution (Dako) followed by cooling down the pressure cooker during 30 min without venting of pressure. Tissue sections were incubated during 30 min at room temperature. Again, the Dako Envision system on the Dako Cytomation autostainer was used for visualisation of the antibody binding and tissue sections were counterstained using haematoxylin and mounted for light microscopy. A tumour sample was regarded positive when at least 10% of all tumour cells on the slide showed nuclear staining.

### cDNA microarrays

cDNA microarrays have been executed as described before (Van Laere *et al*, 2005) on the same patients except for one IBC and two non-IBC breast tumours. High-quality RNA was reverse transcribed, amplified and Cy5 labelled using the Amino Ally MessageAmp aRNA Kit (Ambion Inc., Austin, TX, USA). Universal Human Reference RNA (Stratagene) was processed similarly and Cy3 labelled for competitive hybridisation. cDNA chips were obtained from the Sanger Center and hybridised during 16 h at 47°C in a volume of 40 *μ*l. Information regarding the clone set and the microarray production can be obtained from the World Wide Web: www.sanger.ac.uk/Projects/Microarrays. After hybridisation, slides were washed and scanned immediately using ScanArray software. Data were generated using Quantarray software and analysed using GeneSpring (Agilent Technologies, Palo Alto, CA, USA). The methodology has been described in Van Laere *et al* (2005). Baseline clinicopathologic characteristics for the IBC and non-IBC patients from which samples have been used for cDNA microarray analysis are provided in Table 1.

EGFR, ErbB2, MEK and RAF gene expression signatures, described by Creighton *et al* (2006), were mapped onto our tumour data set using the Locus Link ID resulting in the identification of 54, 55, 50 and 56 common genes respectively. These genes were combined into a MAPK signature of 223 genes represented by 283 clones in our IBC/non-IBC data set (Gene list available as Supplementary information). This MAPK signature was then analysed using Pathway-Express (http://vortex.cs.wayne.edu/projects.htm) to investigate which pathways are over-represented in this gene list. The performance of this MAPK signature to discriminate between ER+ and ER− samples was tested on the Sotiriou data set (Sotiriou *et al*, 2003). Therefore, we submitted the Sotiriou series of 99 breast tissue samples to hierarchical clustering based on the expression of the 223 gene set. Normalised and log 2 transformed expression data for 223 genes were extracted, median-centred on genes and analysed using unsupervised hierarchical clustering with Pearson correlation as similarity metric. Clustering was visualised using GeneSpring.

Next, to investigate common biological themes in our own IBC/non-IBC data set defined by the MAPK signature, unsupervised hierarchical clustering, using centroid linkage clustering with the Pearson correlation coefficient as similarity metric was applied. Global views of the variation in gene expression among the different breast cancer samples defined by the MAPK signature were obtained using principal component analysis on our IBC/non-IBC data set, with the MAPK signature as input data set.

### Statistical analysis

All statistical analysis, except for microarray analyses, have been performed in SPSS (SPSS Inc., Version 12.0, Chicago, IL, USA). Gene expression differences or differences in the percentage of immunostained tumour cell nuclei between two conditions of interest were analysed using the Mann–Whitney *U*-test. Correlations between two binary variables were examined using a Pearson *χ*^2^ test. Correlations between gene expression data were calculated using the Spearman correlation coefficient. Differences or correlations were considered significant when the *P*-value was below 0.05. To investigate common biological themes defined by the expression of ER-*α*, ER-*β*, ER target genes and NF-κB target genes, unsupervised hierarchical clustering has been executed using gene expression data for all NF-κB and ER target genes. Correlations between the clustering patterns and the ER status or the breast tumour phenotype were investigated using a Pearson *χ*^2^ test.

## RESULTS

### Increased activation of NF-κB in ER− breast tumours compared to ER+ breast tumours

**Figure 1:**
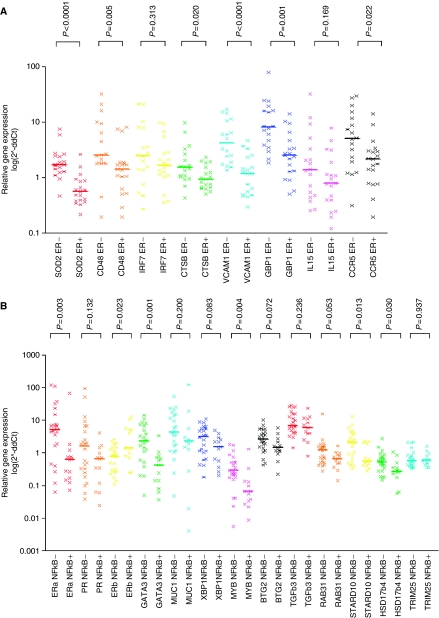
Scatterplot comparing the gene expression levels of NF-κB target genes between ER+ and ER− breast tumour samples (**A**) and scatterplot comparing the gene expression levels of ER target genes between breast tumour samples with and without transcriptionally active NF-κB dimers (**B**). Gene expression data are represented on a log scale. *P*-values for each comparison are displayed on top. The horizontal bars indicate the median gene expression level.

We compared the expression of eight NF-κB target genes in ER+ and ER− breast tumours. Median gene expression levels for all NF-κB target genes were elevated in ER− breast tumours compared to ER+ breast tumours. For 6 out of 8 NF-κB target genes the differences in gene expression between ER+ and ER− breast tumours reached significance. Gene expression data for all NF-κB target genes in ER− and ER+ breast tumours are presented as a scatter plot in Figure 1A. Median gene expression values for the NF-κB target genes in ER− and ER+ breast tumours are indicated by a horizontal bar.

We observed a significant difference between the percentage immunostained nuclei for NFκB1 between ER+ (42.20%) and ER− (61.20%) breast tumours (*P*=0.002) For RelB, a trend towards a significant difference in the percentage of immunostained nuclei was observed (RelB immunostained nuclei in ER+ breast tumours: 29.60%, RelB immunostained nuclei in ER− breast tumours: 52.30%; *P*=0.092). For RelA, cRel and NFkB2, no significant difference was found for the percentage of immunostained nuclei between ER− and ER+ breast tumours. Next, we looked for transcriptionally active NF-κB dimers in ER− and ER+ breast tumours. Therefore, we dichotomised the percentage of immunostained nuclei for each NF-κB transcription factor relative to a cutoff level of 50%. Then, for each sample in our study, we identified transcriptionally active NF-κB dimers by means of examining coexpression of the members of the NF-κB transcription factor family. The detailed methodology was previously described and validated (Van Laere *et al*, 2006b). Hence, we identified 12 out of 18 ER− breast tumours with transcriptionally active NF-κB compared to only 1 out of 19 ER+ breast tumours with transcriptionally active NF-κB (*κ*=−0.609, *P*<0.0001).

### Activation of ER and NF-κB transcription factors is inversely correlated

To further investigate the inverse relationship between NF-κB signalling and ER signalling, the expression of ER-*α*, ER-*β* and 11 putative ER target genes was compared between tumours with and without transcriptionally active NF-κB dimers present. Out of 11 ER target genes, four ER target genes showed significant differences between tumours with and without transcriptionally active NF-κB: GATA3 (*P*=0.001), MYB (*P*=0.004), HSD17*β*4 (*P*=0.030) and STARD10 (*P*=0.013). For XBP1 (*P*=0.083), BTG2 (*P*=0.072) and RAB31 (*P*=0.053) a trend towards significant overexpression was observed. For MUC1 (*P*=0.200), TGF*β*3 (*P*=0.236), TRIM25 (*P*=0.937) and PGR (*P*=0.132) no significant differences were observed. The median gene expression levels were elevated in IHC NF-κB – tumours compared to IHC NF-κB+ tumours for all ER target genes, except TRIM25. In addition, median gene expression levels for ER-*α* were significantly elevated in IHC NF-κB – breast tumours compared to IHC NF-κB+ breast tumours (*P*=0.003). However, gene expression data for ER-*β* was significantly upregulated in IHC NF-κB+ breast tumours compared to IHC NF-κB− breast tumours (*P*=0.023). Gene expression data for different ER target genes in NF-κB+ and NF-κB− breast tumours are displayed in Figure 1B. Median gene expression values for the different ER target genes in NF-κB+ and NF-κB− breast tumours are indicated by a horizontal bar.

**Figure 2:**
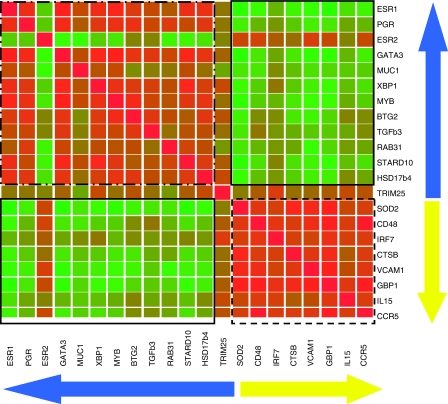
Heatmap comparing gene expression profiles of each gene in the ER/NF-κB signature with each other. Correlation coefficients are colour coded, with red indicating positive correlation coefficients and green indicating negative correlation coefficients. Colour saturation indicates the strength of the correlation coefficients. The blue arrow indicates the ER target genes, the yellow arrow indicates the NF-κB target genes. Two red squares are clearly visible at the points of intersection between the ER target genes on the one hand and the NF-κB target genes on the other, indicating positive correlations (dashed squares). Green squares are visible at the points of intersection between ER and NF-κB target genes, indicating inverse correlations (straight squares).

NF-κB target gene expression data were correlated with gene expression data for ER-*α*, ER-*β* and 11 ER target genes using Spearman correlation coefficients. Data are represented in a heatmap format (Figure 2), in which correlation coefficients are colour coded, with red indicating a positive correlation coefficient and green indicating a negative correlation coefficient. Colour saturation indicates the strength of the correlation coefficient. All NF-κB target genes and most ER target genes are strongly correlated with each other, indicated by the red squares in the heatmap at the point of intersection between the ER target genes on the one hand and NF-κB target genes on the other. Out of 28 comparisons between NF-κB target genes and 78 comparisons between ER-*α*, ER-*β* and 11 ER target genes, respectively 28 and 67 comparisons were positively correlated and in respectively 96 and 62% of the cases, the positive correlation coefficient reached significance (positive Spearman correlation coefficients ranging from 0.285 to 0.879). Interestingly, ER-*β* was anticorrelated with ER-*α* and 7 out of 11 ER target genes, with anticorrelation coefficients reaching significance in four comparisons. Most of the NF-κB and ER target genes are anticorrelated, indicated by the green squares in the heatmap at the point of intersection between the ER and NF-κB target genes. In total, 104 comparisons have been made between ER and NF-κB target genes, from which 77 were anticorrelated and in 40% of the cases the anticorrelation was significant (negative Spearman correlation coefficients ranging from −0.283 to −0.584). These data indicate an inverse correlation between the activation of the NF-κB on the one hand and ER on the other. Gene expression data for ER-*β* were positively correlated with gene expression data for all NF-κB target genes, and correlation coefficients reached significance in five comparisons.

**Figure 3:**
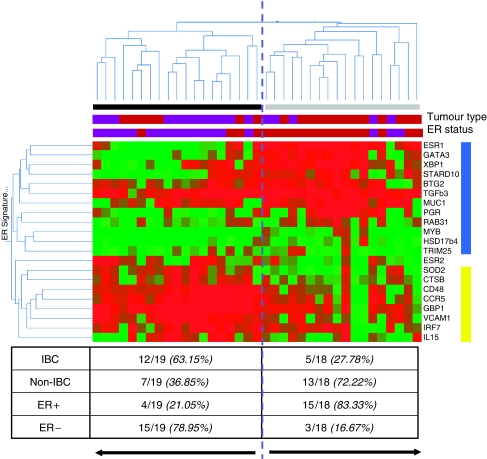
Unsupervised hierarchical clustering using the ER/NF-κB signature. Two clusters are clearly visible, indicated by the black and the grey bar underneath the dendrogram. Gene expression data are represented in matrix format, with rows indicating genes and columns indicating samples. Overexpressed genes are colour coded red and repressed genes are colour coded green. Colour saturation indicates the level of overexpression or repression. The NF-κB signature is indicated by a yellow bar and the ER signature is indicated by a blue bar (right of the matrix). Tumour type (IBC or non-IBC) and ER status (ER+ or ER−) is represented underneath the dendrogram using purple (IBC and ER− respectively) and brown (non-IBC and ER+ respectively) bars. The cluster indicated by the black bar contains 12 out of 19 IBC samples and 15 out of 19 ER− breast tumour samples. The cluster indicated by the grey bar contains 13 out of 18 non-IBC samples and 15 out of 18 ER+ breast tumour samples.

We then applied unsupervised hierarchical clustering to the ER and NF-κB target gene expression data to investigate common biological themes defined by the combined ER and NF-κB signature. The clustering output is displayed in Figure 3. Two major sample clusters have been identified, as well as two major gene clusters. The gene clusters represented the NF-κB target gene signature on the one hand and the ER target gene signature on the other hand. A first sample cluster was characterised by an increased expression of the NF-κB signature and a decreased expression of the ER signature. A second sample cluster was characterised by an increased expression of the ER target gene signature and a decreased expression of the NF-κB target gene signature. The first sample cluster was characterised by an increased amount of IBC samples. Out of 17 IBC samples, 12 IBC samples belonged to the NF-κB positive cluster whereas five IBC samples belonged to the NF-κB negative cluster. Out of 20 non-IBC samples, 13 non-IBC samples belonged to the NF-κB negative cluster and seven non-IBC samples belonged to the NF-κB positive cluster (Pearson *χ*^2^; *P*=0.001). However, when looking at the ER status, out of 19 samples in cluster 1, 15 samples were identified as being ER−. Out of 18 samples in cluster 2, again 15 samples were identified as being ER+ (Pearson *χ*^2^; *P*<0.0001), indicating that elevated NF-κB activation is more common in ER− breast tumours, independent of the IBC of non-IBC phenotype and that the frequent activation of NF-κB in IBC is caused by the frequent ER negativity of IBC specimens.

### EGFR/ErbB2-MAPK hyperactivation leads to NF-κB activation and ER downregulation

The MAPK signature composed of 223 genes up- or downregulated in MCF7 cell lines, after transfection with EGFR (+EGF), ErbB2, RAF and MEK was first analysed using Pathway-Express to investigate which signal transduction pathways are represented in this gene list. Not surprisingly, the MAPK pathway was most significantly linked to the MAPK signature (Rank 1, *P*<0.001). However, other important signal transduction pathways were also represented in the MAPK signature among which: Wnt signalling pathway (Rank 5, *P*=0.005), Notch signalling pathway (Rank 6, *P*=0.006), VEGF signalling pathway (Rank 7, *P*=0.008) and the Toll-like receptor signalling pathway (Rank 10, *P*=0.009).

**Figure 4:**
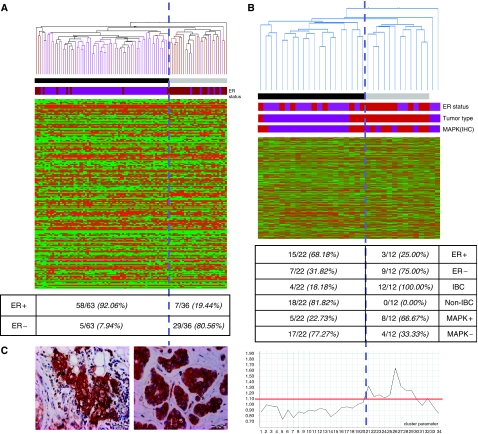
The performance of the MAPK signature for prediction of ER status was validated using the Sotiriou data set (**A**). Unsupervised hierarchical clustering was executed. Data are represented in matrix format, with rows indicating genes and columns indicating samples. Overexpressed genes are colour coded red and repressed genes are colour coded green, missing values are colour coded grey. Colour saturation indicates the level of overexpression or repression. Two clusters have been identified indicated by the black and grey bars underneath the dendrogram. ER status is represented by the purple (ER+) and brown (ER−) bar. The MAPK signature was then applied onto our own IBC/non-IBC expression data set using unsupervised hierarchical clustering (**B**). Again, data are represented in a matrix format, with rows representing genes and columns representing samples. Overexpressed genes are colour coded red and repressed genes are colour coded green. The level of overexpression or repression is represented by the colour saturation. Two clusters have been identified indicated by the black and grey bars underneath the dendrogram. Tumour type (IBC or non-IBC), ER status (ER+ or ER−) and MAPK status (presence or absence of EGFR and/or ErbB2 overexpression) are indicated using brown (IBC, ER− and presence of EGFR and/or ErbB2 overexpression respectively) and purple (non-IBC, ER+ and absence of EGFR and/or ErbB2 overexpression respectively) bars. The cluster indicated by the black bar contains 15 out of 22 ER+ breast tumour samples, 18 out of 22 non-IBC specimens and 17 out of 22 MAPK-negative breast tumour specimens. The cluster indicated by the grey bar contains 9 out of 12 ER− breast tumour samples, 12 out of 12 IBC specimens and 8 out of 12 MAPK+ breast tumour specimens. The expression profile of the first metagene, representing approximately 44% of the total variation seen within this data set, is shown underneath the matrix. The red line indicates a relative gene expression level of 1 (reference). Microphotographs showing membranous staining for EGFR (left) and ErbB2 (right) are displayed in (**C**).

Next, we tested the MAPK signature for performance by unsupervised hierarchical clustering on the Sotiriou data (Sotiriou *et al*, 2003) set to identify ER status. The output of the clustering analysis is shown in Figure 4A. We identified one large cluster that was mainly composed of ER+ specimens (58 out of 63) and two smaller clusters mainly composed of ER− specimens (29 out of 36) (Pearson *χ*^2^, *P*<0.0001). In total, the performance of the MAPK signature in predicting the ER status by unsupervised analysis of the Sotiriou data set (Sotiriou *et al*, 2003) was 88%. This clearly demonstrated that the MAPK signature is capable of predicting ER status.

Next, the MAPK signature was applied onto our own IBC/non-IBC data set (Van Laere *et al*, 2005) by unsupervised hierarchical clustering analysis. The output of the clustering analysis is shown in Figure 4B. We identified two clusters, one which was mainly enriched in ER− breast cancer specimens (9 out of 12) and a second which was mainly enriched in ER+ breast cancer specimens (15 out of 22) (Pearson *χ*^2^, *P*<0.001), resulting in an accurate prediction of IHC ER status in 71% of the cases. However, when looking at the distribution of the different IBC/non-IBC samples over the different clusters, we found that the first cluster was exclusively composed of IBC specimens (12 out of 12), whereas the second cluster was mainly composed of non-IBC specimens (18 out of 22) (Pearson *χ*^2^, *P*<0.0001), resulting in an accurate prediction of the tumour phenotype in 88% of the cases. We then performed a principle component analysis with the MAPK signature as input data set to obtain global views of the variation in the IBC/non-IBC data set, defined by the MAPK signature. We identified that the expression of the first metagene, being the first principal component generated by the principal component analysis, accounting for approximately 44% of the total variation seen in this data set, was significantly overexpressed in IBC (median: 1.127) compared to non-IBC (median: 0.904) (*P*<0.001). The expression profile of the first metagene is drawn underneath the clustering output in Figure 4B. The red line indicated a relative gene expression level of 1. Most IBC specimens have a strongly elevated expression for the first metagene (above 1) whereas most non-IBC specimens have a reduced expression for the first metagene (beneath 1).

Next, we analysed if the samples with a strong expression for the first metagene were also characterised by an overexpression of EGFR and/or ErbB2. Therefore, we performed IHC for EGFR and ErbB2 (Figure 4C) and scored membranous staining. IHC data were then mapped onto the dendrogram and the results are shown in Figure 4B. Most samples characterised by an elevated expression for the first metagene were also characterised by an overexpression of EGFR and/or ErbB2 as shown in Figure 4B. The expression of the first metagene was significantly elevated in tumours characterised by EGFR/ErbB2 overexpression (median: 1.109) compared to tumours without overexpression of EGFR or ErbB2 (median: 0.909) (*P*=0.004).

Finally, IHC data for EGFR and/or ErbB2 overexpression were correlated with tumour phenotype (IBC/non-IBC), ER status and the presence of transcriptionally active NF-κB dimers. The presence of membranous staining for EGFR and/or ErbB2 was significantly correlated with tumour phenotype. We identified 13 out of 17 IBC tumours with EGFR and/or ErbB2 overexpression compared to only one non-IBC tumour with EGFR and/or ErbB2 overexpression (*κ*=0.742, *P*<0.0001). Out of 13 samples with transcriptionally active NF-κB dimers, nine showed overexpression of EGFR and/or ErbB2 whereas out of 24 tumours without transcriptionally active NF-κB dimers only five showed overexpression of EGFR and/or ErbB2 (*κ*=0.476, *P*=0.004). The overexpression of EGFR and/or ErbB2 was also significantly anticorrelated with ER status. Out of 19 ER+ breast tumours 15 showed no overexpression of EGFR and/or ErbB2 whereas 10 out of 18 ER− breast tumours showed overexpression of EGFR and/or ErbB2 (*κ*=−0.343, *P*=0.031). Using tumour phenotype (IBC or non-IBC), ER expression and presence of transcriptionally active NF-κB as independent variables and EGFR and/or ErbB2 overexpression as dependent variable, a logistic regression was performed. Tumour phenotype was identified as the most predictive parameter to discriminate between tumour samples with EGFR and/or ErbB2 overexpression and without EGFR and ErbB2 overexpression (*β*=18.039; *P*=0.038). The presence of transcriptionally active NF-κB (*β*=7.720; *P*=0.092) and expression of ER (*β*=−6.800; *P*=0.122) were both not significantly associated with EGFR and/or ErbB2 overexpression in this model.

## DISCUSSION

The activation of NF-κB has been described previously in human breast cancer and breast cancer cell lines and has been specifically linked to ER independency. Biswas *et al* reported that NF-κB is activated more often in ER− human breast tumours compared with ER+ breast tumours and most predominantly in ER− and ErbB2+ breast tumours (Biswas *et al*, 2004). Zhou *et al* studied the activation of NF-κB in two groups of ER+ breast tumours. Early-stage primary breast cancer selected for lower ER content showed two- to fourfold increased NFkB1 and RelA DNA binding over a second set of primary breast cancer with higher ER content. This demonstrates that the level of NF-κB activation is inversely correlated with ER content (Zhou *et al*, 2005). In a recent study, we described that the expression of several NF-κB target genes was significantly elevated in IBC compared to non-IBC (Van Laere *et al*, 2006b). In addition, the increased activation status of NF-κB in IBC was confirmed by immunohistochemistry and DNA-binding experiments. Considering the fact that the activation of NF-κB is generally linked to ER independency, these findings can be explained by the fact that, compared to non-IBC, IBC has a low frequency of ER+ tumour specimens. In this study, we show that the activation of NF-κB is indeed more generally linked to breast tumours without ER protein expression than specifically to the IBC phenotype. Breast tumours with transcriptionally active NF-κB show reduced expression of most ER target genes, whereas ER+ breast tumours show reduced expression of most NF-κB target genes. The expression of ER-*α* and most ER target genes is anticorrelated with the expression of most NF-κB target genes in our data set of 37 IBC and non-IBC breast tumours, indicating an inverse correlation between ER and NF-κB activation. By means of unsupervised hierarchical clustering using the combined ER and NF-κB data set we identified two sample clusters, one of which was characterised by a low expression of ER target genes and high expression of NF-κB target genes, and a second with high expression of ER target genes and low expression of NF-κB target genes. The distribution of the different tumour samples over both clusters showed that the activation of NF-κB was not exclusively linked to the IBC phenotype, but more generally to breast tumours without ER expression at protein level.

Several mechanisms resulting in the inverse correlation between NF-κB activation and ER activation have been proposed (Kalaitzidis and Gilmore, 2005). ER has been shown to block NF-κB activity at several steps: (a) it can inhibit the IKK activity; (b) it can inhibit degradation of I*κ*B; (c) it can block DNA binding by NF-κB; (d) it can bind coactivators and compete with NF-κB for coactivators binding; and (e) it can directly bind to DNA-bound NF-κB to inhibit NF-κB-mediated transcriptional activation. In addition, the cross-coupling between ER and NF-κB also results in reduced activity of promoters with ER binding sites (Stein and Yang, 1995). The inverse correlation between NF-κB activation and ER activation can also be explained alternatively. Recent evidence suggests that all ER− breast tumours arise from ER+ breast tumour cells that stop expressing ER-*α* (Creighton *et al*, 2006). One of the reasons for the downregulation of ER-*α* is the overexpression of EGFR and/or ErbB2 resulting in the hyperactivation of MAPK (Oh *et al*, 2001). This mechanism possibly involves the activation of NF-κB (Holloway *et al*, 2004). The importance of hyperactivated MAPK for the generation of ER− breast tumours has been demonstrated by the fact that a MAPK signature is able to discriminate between ER+ and ER− breast tumours in four independent data sets with accuracies ranging from 68 to 87% (Creighton *et al*, 2006). The application of a MAPK signature to our own IBC/non-IBC data set resulted in the correct prediction of ER status in 70% of the cases. However, the separation between IBC and non-IBC samples, based on the MAPK signature, was even more strict, with almost all IBC specimens falling together in one cluster and almost all non-IBC specimens falling together in a second cluster, independent of ER status. The presence of the VEGF, Wnt, Notch and Toll-like Receptor signalling pathways in the MAPK signature may account for the clustering pattern. Previously, it has been extensively demonstrated that IBC is characterised by increased angiogenesis (Van der Auwera *et al*, 2004) for which VEGF signalling is very important. Additionally, microarray experiments have shown that Wnt and Notch signalling pathways are represented in an IBC gene expression signature (Van Laere *et al*, 2005). In addition, the Toll-like Receptor signalling pathway, also linked to the IBC gene expression signature (Van Laere *et al*, 2005), leads to the activation of NF-κB, a hallmark of IBC (Van Laere *et al*, 2006b). Altogether, this clustering pattern again underscores the distinct biological phenotype of IBC. Additionally, these results agree with our findings that IBC more often displays features of the Basal-like or ErbB2-overexpressing cell-of-origin breast cancer subtypes (Van Laere *et al*, 2006a) as described by Perou *et al* (2000). Breast tumours belonging to these subclasses more often demonstrate respectively EGFR and ErbB2 overexpression (Nielsen *et al*, 2004).

From the expression of the first metagene in IBC and non-IBC we learned that IBC is characterised by a more frequent MAPK hyperactivation, which is reflected by the frequent overexpression of EGFR and/or ErbB2 in IBC compared to non-IBC. Previous studies have shown, that the MAPK pathway is involved in RhoC GTPase induced motility, invasion and angiogenesis in IBC (Van Golen *et al*, 2002). Other studies have shown that the stimulatory effect of the RhoC GTPase-activity on tumour growth depends on NF-κB activity (Benitah *et al*, 2003). One of the first molecular alterations characterised in IBC was the overexpression of RhoC (Van Golen *et al*, 1999). In combination with our data, this suggests that IBC is generally characterised by overexpression of EGFR and/or ErbB2, leading to MAPK-induced activation of NF-κB, which then in turn results in RhoC overexpression and loss of ER expression. However, these associations have only been marginally underscored using a multivariate regression model, except for the association between tumour phenotype and EGFR and/or ErbB2 overexpression. This indicates that more elaborate research using a larger amount of breast tumours is needed to confirm the observed associations. In addition, it should be noted that our findings have been based upon correlations between gene expression and protein expression data. Further investigation, using IBC and non-IBC cell lines, should be performed to mechanistically confirm the proposed interactions. Nevertheless, our data are in close agreement on previously published research on NF-κB activation in breast cancer, both in cell lines and in human samples (Biswas *et al*, 2000; Zhou *et al*, 2005).

The absence of NF-κB activation in ER+ cancers remains remarkable, and is explained by specific inhibitory crosstalk between NF-κB and ER signalling. The precise intersection of ER with the NF-κB pathway is not clear, and may be at multiple points (as discussed above). The specific inactivation of NF-κB signalling by ER and oestrogen implies a specific mechanism that is selected by ER+ breast cancers. During endocrine therapy, resistance invariably develops. For agonist–antagonist drugs, such as Tamoxifen, a shift from antagonist to agonist activity appears to be an important mechanism of resistance. For newer aromatase inhibitors, which deprive tumours of oestrogen, resistance mechanisms are not yet described. NF-κB activation, through escape from tonic inhibition by oestrogen, is a reasonable candidate mechanism for resistance to oestrogen deprivation. Hence, as suggested by our data, NF-κB can be an important therapeutic target for patients treated with aromatase inhibitors developing resistance to oestrogen deprivation as well as for patients having EGFR and/or ErbB2 overexpression.

In conclusion, the MAPK signature performs better in separating IBC from non-IBC tumours than in distinguishing ER− from ER+ breast tumours. This despite the clearly demonstrated performance of the MAPK signature in distinguishing ER− from ER+ breast tumours in the Sotiriou data set. The elevated expression of the MAPK signature, characterizing IBC, is reflected by the high percentage of EGFR and/or ErbB2 overexpressing IBC tumours, which potentially leads to the frequent ER independency of IBC, as well as to an increased activation of NF-κB, accounting for the inverse interaction between NF-κB and ER activation. It should be noted that, the increased activation of NF-κB is not exclusively linked to the IBC phenotype, but more generally to ER-independent breast tumours, which have a more frequent EGFR and/or ErbB2 amplification, hence breast tumours belong to the Basal-like and ErbB2-overexpressing cell-of-origin subtypes respectively. The relationship between EGFR and/or ErbB2 overexpression, MAPK hyperactivation, NF-κB transcriptional activity and loss of ER protein expression in Basal-like or ErbB2-overexpressing breast tumours is currently under investigation.

## Figures and Tables

**Table 1:** Clinicopathological characteristics for the study population

	**PCR (*n*=37)**		**cDNA array (*n*=34)**	
	**Non-IBC (*n*=20)**	**IBC (*n*=17)**	**Non-IBC (*n*=18)**	**IBC (*n*=16)**
*Age (years)*				
Median (range)	61 (42–78)	56 (41–74)	61 (42–78)	56 (41–74)
				
*Histological type*				
Ductal	18	15	16	14
Lobular	2	2	2	2
				
*Tumour emboli in dermal lymph vessels*				
Present	2	14	2	13
Absent	18	3	16	3
				
*Grade* a				
1	6	0	5	0
2	8	8	8	7
3	6	9	5	9
				
*T-Stadium*				
1	6	0	5	0
2	4	0	3	0
3	7	0	7	0
4	3	17	3	16
				
*N-Stadium* b				
0	8	0	7	0
1	11	6	10	5
2	1	11	1	11
				
*ER-status* c				
ER negative	5	13	4	12
ER positive	15	4	14	4
				
*PR-status* d				
PR negative	9	14	7	13
PR positive	11	3	11	3

IBC=inflammatory breast cancer.

a According to the Elston–Ellis modification of the SBR grading system.

b The N-Stadium for patients with IBC was determined clinically.

c ER status determined using the anti-ER antibody (PharmDX) and a cutoff level of 10% to discriminate between absence or presence of nuclear protein expression.

d PR status determined using the anti-PR antibody (clone PgR1249) and a cutoff level of 10% to discriminate between absence or presence of nuclear protein expression.
